# Patient-Specific Cell Communication Networks Associate With Disease Progression in Cancer

**DOI:** 10.3389/fgene.2021.667382

**Published:** 2021-08-27

**Authors:** David L. Gibbs, Boris Aguilar, Vésteinn Thorsson, Alexander V. Ratushny, Ilya Shmulevich

**Affiliations:** ^1^Institute for Systems Biology, Seattle, WA, United States; ^2^Bristol Myers Squibb, Seattle, WA, United States

**Keywords:** systems biology, bioinformatics, computational oncology, immuno-oncology, cell communication, networks

## Abstract

The maintenance and function of tissues in health and disease depends on cell–cell communication. This work shows how high-level features, representing cell–cell communication, can be defined and used to associate certain signaling “axes” with clinical outcomes. We generated a scaffold of cell–cell interactions and defined a probabilistic method for creating per-patient weighted graphs based on gene expression and cell deconvolution results. With this method, we generated over 9,000 graphs for The Cancer Genome Atlas (TCGA) patient samples, each representing likely channels of intercellular communication in the tumor microenvironment (TME). It was shown that cell–cell edges were strongly associated with disease severity and progression, in terms of survival time and tumor stage. Within individual tumor types, there are predominant cell types, and the collection of associated edges were found to be predictive of clinical phenotypes. Additionally, genes associated with differentially weighted edges were enriched in Gene Ontology terms associated with tissue structure and immune response. Code, data, and notebooks are provided to enable the application of this method to any expression dataset (https://github.com/IlyaLab/Pan-Cancer-Cell-Cell-Comm-Net).

## Introduction

The maintenance and function of tissues depends on cell–cell communication (Wilson et al., 2000; Haass and Herlyn, 2005). While cell communication can take place through physically binding cell membrane surface proteins, cells also release ligand molecules that diffuse and bind to receptors on other cells (paracrine or endocrine), or even the same cell (autocrine), triggering a signaling cascade that can potentially activate a gene regulatory program (Cameron and Kelvin, 2013; Heldin et al., 2016; Cohen and Nelson, 2018). Cells communicate to coordinate activity, such as correctly (and jointly) responding to environmental changes (Song et al., 2019). More generally, a message is sent and received, transferring some information as part of a large network (Frankenstein et al., 2006).

Altered cellular communication can cause disease, and conversely diseases can alter communication (Wei et al., 2004). Cancer, once thought of as purely a disease of genetics, is now recognized as being enmeshed in complex cellular interactions within the tumor microenvironment (TME; Trosko and Ruch, 1998). The cell–cell interactions are shown to be important for cell differentiation, tumor growth (West and Newton, 2019), and response to therapeutics (Kumar et al., 2018).

One approach to studying cell interactions is through the use of graphical models of communication networks (Morel et al., 2017). By incorporating experimental data, the graphical models can become quantitative, providing predictions that can be tested and used in discovering novel drug targets and developing optimal intervention strategies.

In recent work, Thorsson et al. (2018) developed a method used to identify cellular communication networks. Given a set of samples, the method identified ligands, receptors and cells meeting certain criteria of abundance and concordance. The method was applied in the identification of networks that play a role within specific tumor types and molecular subtypes and is available as a workflow and interactive module on the CRI-iAtlas portal for immuno-oncology (Eddy et al., 2020).

**FIGURE 1:**
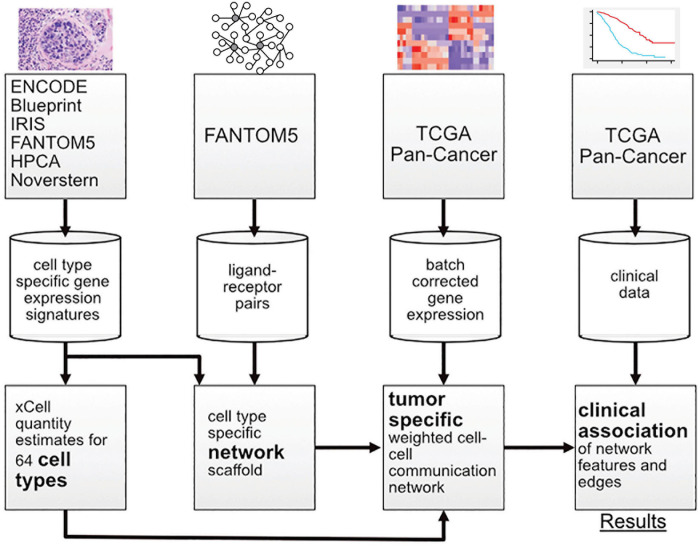
Overview of the workflow showing the transition from data sources to results.

In this work, multiple sources of data were integrated with a new probabilistic method for constructing patient-specific cell–cell communication networks (Figure 1). In total, we built networks for 9,234 samples in The Cancer Genome Atlas (TCGA), starting from a network of 64 cell types and 1,894 ligand-receptor pairs and we identified informative network features that are associated with disease progression. The method can be applied to any cancer type, but in this manuscript we focus on a selection of cancer types with high mortality rates, including pancreatic adenocarcinoma (PAAD), melanoma (SKCM), lung squamous cell carcinoma (LUSC), and cancers of the gastrointestinal tract (ESCA, STAD, COAD, and READ) (Cancer Genome Atlas Network, 2015).

This represents a new method that provides information on possible subtypes of intercellular signaling in the TME, something that is currently lacking. While there are many methods of gene set scoring, cellular abundance estimation, and differential expression, there are still few ways to investigate cell–cell communication diversity in the TME with respect to patient outcomes. Fortunately, new databases of receptor-ligand pairs are becoming available, making work in this area possible (Efremova et al., 2019; Jin et al., 2020; Nath and Leier, 2020; Shao et al., 2020). The methods, code, data, and complete results are available and open to all researchers^1^.

## Materials and Methods

### Data Aggregation and Integration

Data sources including TCGA and cell-sorted gene expression, bulk tumor expression, cell type scores, cell–ligand and cell–receptor presence estimations were used for network construction and probabilistic weighting on a per-sample basis.

Each tumor sample is composed of a mixture of cell types including tumor, immune, and stromal cells. Recently, methods have been developed to “deconvolve” mixed samples into estimated fractions of cell type quantities. xCell, a variation on this theme, has gene set enrichment like scores available for 64 cell types across most TCGA samples (Aran et al., 2017). While many cell deconvolution and scoring methods exist, xCell provides a wide array of cell types including immune and stromal cells that are not provided in other methods. xCell uses six public cell sorted bulk gene expression data sets to generate gene signatures and score each TCGA sample. In practice, this means that each cell type has a set of genes associated with it, a gene signature, which is used in producing a numerical score related to the quantity of that cell type in the sample. Across the primary data sets, there is some discrepancy in cell type nomenclature, making it necessary to manually curate cell type names to facilitate integration across experiments (Supplementary Table 1). Typically, for a given cell type, there are several replicate expression profiles, both within and across data sets.

Regarding a map connecting cells via ligands and receptors, Ramilowski et al. (2015) performed a comprehensive survey of cellular communication, generating a compendium that includes 1,894 ligand-receptor pairs, a mapping between 144 cell types, and expression of ligand or receptor molecules. The compendium was shared via the 5th edition of the FANTOM Project, FANTOM5. These ligand-receptor pairs were adopted for this study. Unfortunately, the FANTOM5 collection of cell types does not overlap well with cell types in xCell. In order to integrate the xCell and FANTOM5 data resources, it was necessary to determine the expressed ligands and receptors for each of the 64 cell types in xCell, using the source gene expression data.

### Building the Cell-Cell Communication Network Scaffold

In the FANTOM5 “draft of cellular communication,” an expression threshold of 10 TPMs was used to link a cell type to a ligand or receptor. When considering the distribution of expression in the FANTOM5 project, 10 TPMs is close to the median.

To construct our scaffold, we used a majority voting scheme based on comparing expression levels to median levels. For each cell type, paired with ligands and receptors, if the expression level was greater than the median, it was counted as a vote (i.e., ligand expressed in this cell type). If a ligand or receptor receives a majority vote across all available data sources, it was accepted, and entered into the cell–cell scaffold.

With this procedure, a network scaffold is induced, where cells produce ligands that bind to receptors on receiving cells. One edge in the network is composed of components cell–ligand–receptor–cell. This produces a cell-cell communication network with over 1M edges. Each edge represents a possible interaction in the TME. We subsequently determine the probability that an edge is active in a particular patient sample using a probabilistic method described below.

### Patient Level Cell-Cell Communication Network Weights

**FIGURE 2:**
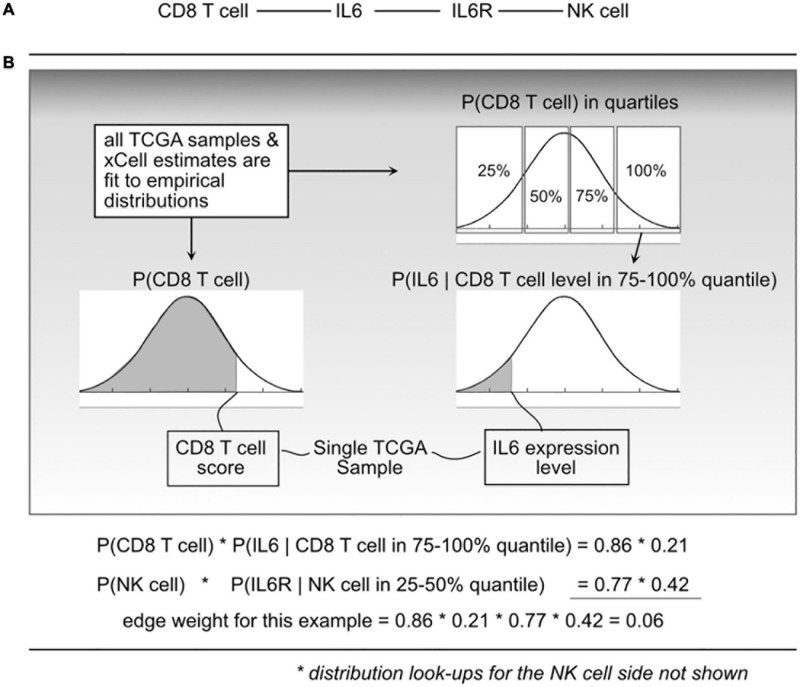
Illustration of the probabilistic model and edge weight computations. **(A)** For a given cell-cell communication edge, **(B)** per patient values are used to “look up” probabilities from the distributions learned from all The Cancer Genome Atlas (TCGA) data. Those probabilities are then used to compute an edge weight.

With a cell–cell scaffold, expression values and cell type scores per sample, we can produce a per-sample weighted cell–cell communication network (Figure 2). This is done probabilistically, using the following definition:

(1) $$P\,\left(e_{i}\right)=P\,\left(l_{a},c_{l}\right)\cdot P\,\left(r_{b},c_{r}\right),$$

where *e_i* is edge *i*, *l_a* is ligand *a*, *r_b* is receptor *b*, and *c_l* and *c_r* are cells that can produce ligand *a* and receptor *b*, respectively. *P*(*e*_*i*_) represents a probability that edge *i* is active based on the premise that the physical and biochemical link and activation is possible only if all the components are present, and that activity becomes increasingly possible with greater availability of those components. The joint probabilities can be decomposed into:

(2) $$P\,\left(l_{a},c_{l}\right)=P\,\left(l_{a}|c_{l}\right)\,P\,\left(c_{l}\right)\,\text{and}$$

$$P\,\left(r_{b},c_{r}\right)=P\,\left(r_{b}|c_{r}\right)\,P\,\left(c_{r}\right)$$

The *P*(*c*_*lk*_) is short for CDF *P*(*C*_*l*_ < *c*_*lk*_) which indicates the probability that a randomly sampled value from the empirical *C_l* distribution (over all 9K TCGA samples) would be less than the cell estimate for cell type *l*, in sample *k*. To do this, for a given cell type, using all samples available, an empirical distribution *P*(*C*_*l*_) is computed, and for any query, essentially using a value *c*_*lk*_, the probability can be found by integrating from 0 to *c*_*lk*_.

To compute *P*(*l*_*ak*_|*c*_*lk*_) and *P*(*r*_*bk*_|*c*_*rk*_), each *C_l* and *C_r* distribution was divided into quartiles, and then (again using the 9K samples) empirical gene expression distributions within each quartile were fit. This expresses the probability that with an observed cell quantity (thus within a quartile), the probability that a randomly selected gene expression value (for gene *l_a*) would be lower than what is observed in sample *k*.

We refer to “edge weights” to be the probability *P*(*e*_*i*_) as shown in Eq. 1. To compute edge weights, each TCGA sample was represented as a column vector of gene expression and a column vector of cell quantities (or xCell scores). For each edge in the scaffold (cell–ligand–receptor–cell), probabilities using the defined empirical distributions based on sample values and then taking products for the resulting edge weight probability. This leads to over 9K tumor-specific weighted networks, one for each TCGA participant.

Probability distributions were precomputed using the R language empirical cumulative distribution function (ecdf). For example, fitting *P*(CD8 T cells) is done by taking all available estimates across the Pan-Cancer samples and computing the ecdf. Then, for a sample *k*, we find *P*(*C*_*l*_ < *c*_*lk*_) using the ecdf. The same technique is used to find the conditional probability functions, where for each gene, the expression values are selected after binning samples using the R function “quantile,” and then used to compute the ecdf. With all distributions precomputed, 9.8 billion joint probability functions were computed using an HPC environment, then transferred to a Google BigQuery table where analysis proceeded. This table of network weights was structured so that each row contained one weight from one edge and one tumor sample. Although being a large table of 9.8 billion rows, taking nearly 500GB, BigQuery allows for fast analytical queries that can produce statistics using a selection of standard mathematical functions.

### Association of Network Features and Survival-Based Phenotypes

As an initial examination of the interplay of cell communication and disease, two proxies of disease severity were investigated: progression-free interval (PFI) and tumor stage (Liu et al., 2018). The staging variable used the American Joint Committee on Cancer (AJCC) pathologic tumor stages. The PFI feature was computed using days until a progression event. The staging variable was binarized by binning stages I–II together (“early stage”), and III–IV together (“late stage”). A binary PFI variable was created by computing the median PFI on non-censored samples and then applying the split to all samples. Both clinical features were computed by tissue type (TCGA Study). As Liu et al. writes, “The event time is the shortest period from the date of initial diagnosis to the date of an event. The censored time is from the date of initial diagnosis to the date of last contact or the date of death without disease.”

For example, in LUSC, the median time to PFI event was 420 days (14 months) and in the censored group, 649 days (21.1 months). After splitting samples at 420 days (14 months), the short PFI group was composed of 67 uncensored samples and 128 censored samples. The long PFI group was composed of 68 uncensored samples and 234 censored samples.

**FIGURE 3:**
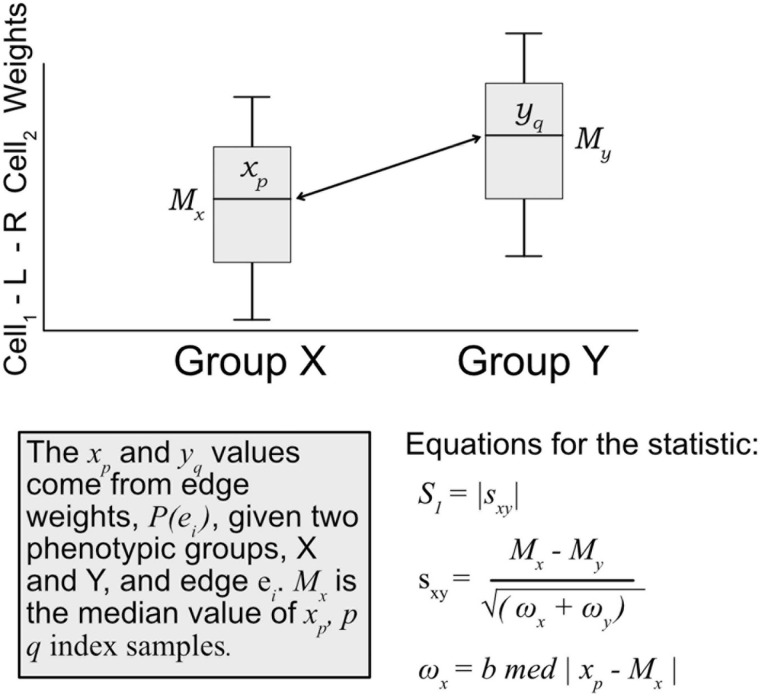
Diagram of how the *S*_1_ statistic is calculated. Given two groups and one edge of interest, the statistic is generated using edge weights as input. For each group, both the median and maximum absolute deviation of edge weights are calculated and used to compute the S_1_. The resulting robust statistic describes the magnitude of difference between groups for one edge.

A modified *S*_1_ statistic, a robust measure of differences, can be used for comparing phenotypic groups (Babu et al., 1999; Yahaya et al., 2004; Hubert et al., 2012; Ahad et al., 2016). The modified forms of the S_1_ statistic are shown to better control type 1 errors. Here, the statistic is calculated as

(3) $$S_{1}=\left|s_{x\,y}\right|$$

$$s_{x\,y}=\frac{M_{x}-M_{y}}{\sqrt{\omega_{x}+\omega_{y}}}$$

$$\omega_{x}=b\,m\,e\,d\,\left|x_{p}-M_{x}\right|$$

where *M*_*x*_ is the sample median of edges weights [*P*(*e*_*i*_) Eq. 1] for a given edge *e*_*i*_ in phenotypic group x, likewise for phenotypic group y with *M*_*y*_. ω_*x*_ is defined as the median absolute deviation (MAD) on edge weights, *x*_*p*_ ∈ *P*(*e*_*i*_), for phenotypic group *x*, and likewise for group y with ω_*y*_ (see Figure 3). For *b*, the MAD default constant of 1.4826 is used. The *S*_1_ statistic is defined as the absolute value of *s*_*xy*_, but since we are interested in the directionality of the value, we mostly are concerned with s_*xy*_, and use the absolute value when considering whether an edge should be designated as “high value.” Since each edge is scored this statistic allowed for cell–cell interactions to be ranked within a defined context. Per tissue type *S*_1_ statistics were computed using the ISB-CGC Cloud Resource. The implementation of the statistic was written in BigQuery SQL and the results were again saved to BigQuery tables to allow for further cloud-based analysis and integration with underlying data.

**FIGURE 4:**
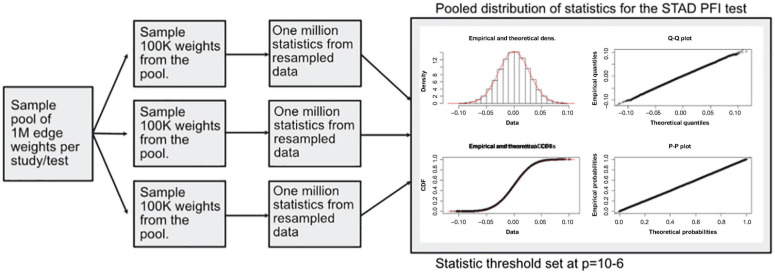
Diagram of the resampling procedure for calculating differentially weighted edges. Three sample pools of edge weights were taken from each tissue source. Then matching the sample sizes for clinical features, edge weight samples were used in computing randomized *S*_1_ statistics. For each sample pool, tumor type, and phenotype combination, 1 million statistics were produced, and the millionth percentile on combined statistics (3M total) was used as a cutoff in determining important edges.

A modified bootstrap procedure was used to judge the magnitude of the statistic with respect to a random sample (Figure 4). An ensemble of three edge-weight sample-pools were sampled from existing values, each with 100K weights. Then, for each member of the ensemble, 1 million *S*_1_ statistics were generated using sample sizes that match the analyzed data. These random *S*_1_ statistic distributions were used to compare to the observed results. The random *S*_1_ distributions were close to Normal with heavy tails (Supplementary Figure 1); the locations were near zero but with varying scales (Supplementary Figure 3). After combining resampled statistics across the ensemble, an edge was designated as a high value edge if the absolute value S_1_ was in the top 1 millionth percentile of the absolute value random *S*_1_ distribution. Each tissue and contrast (comparison between PFI groups) generates a weighted subgraph of the starting scaffold, which is retained for further analysis (e.g., a LUSC-PFI network).

Using high value edges, informative cell-cell interactions that relate to disease progression were identified. To do this, machine learning models were trained on binarized clinical data as described with PFI and tumor stage as the response or target. A matrix of patient-specific edge weight predictors for each tissue was constructed. Classification of samples was performed with XGBoost classifiers (Chen and Guestrin, 2016), which are composed of an ensemble of tree classifiers. To avoid overfitting the models, the tree depth was set at maximum of 2 and the early-stopping parameter was set at 2 rounds (training was stopped after the classification error did not improve on a test set for two rounds). XGBoost provides methods for determining the information gain of each feature in the model and was used to rank edges that are most informative for classification.

Gene ontology (GO) term enrichment was performed using the GONet tool (Pomaznoy et al., 2018). The set of 1,175 genes in the cell-cell scaffold was used as the enrichment background. GONet builds on the “goenrich” software package, which maps genes onto terms and propagates them up the GO graph, performs Fisher’s exact tests, and moderates results with FDR. To compare the results, random collections of genes were generated from the cell-cell scaffold and produced no significant results.

## Results

In the scaffold graph, a cell produces a ligand that binds a receptor found on another cell type, which could make a single edge “LCell–Ligand–Receptor–RCell.” In total, there were 1,062,718 cell–cell edges in the network. The number of expressed ligands and receptors varied by cell type. For ligand-producing cells this ranged from 32,910 for osteoblasts to 6,587 for Multi-Potent Progenitor (MPPs). For receptor-producing cells, the range spans from 30,225 for platelets to 5,763 edges for MPPs.

Applying the proposed probabilistic framework allowed for the creation of 9,234 weighted networks. The edge weight distributions generally follow approximately an exponentially decreasing function (Supplementary Figure 1). There are few edges with strong weights and many with near zero weights.

**FIGURE 5:**
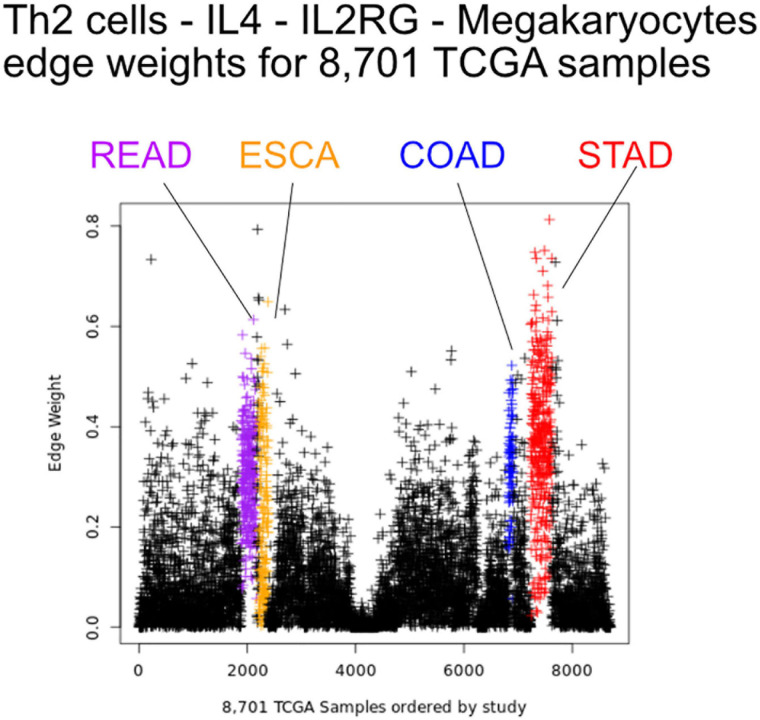
Edge weights can distinguish tissue types. Each point represents a tumor sample, sorted by tissue type. Shown is the Th2 cell-IL4-IL2RG-Megakaryocytes edge colored by tissue source: STAD red, READ blue, COAD purple, and ESCA orange.

We first sought to find communication edges that were most characteristic of an individual tumor type. The *S*_1_ statistics comparing one tissue to all other tissue types was computed, with a high score indicating a substantial difference in edge weights between the two groups. Edges were found that clearly delineated tissues (Figure 5). For example, in skin cutaneous melanoma (SKCM), the top scoring edge is between melanocytes, the usual cell of origin for cutaneous melanoma (Melanocytes-MIA-CDH19-Melanocytes, S*_1_* score 2.5, median edge weight 0.86 higher than in other tumor types). Normal tissue differences can contribute to differences in edge weights, though in this case the central role of melanocytes in melanomas implies that the high scores are likely due to cancerous cell activity. Similarity is seen with the uveal melanoma (UVA) study, where cancer likely stems from melanocytes resident in the uveal tract (Robertson et al., 2017). Additionally, we observed that when a cell type is highly prevalent in a particular tissue, and the scaffold has an autocrine loop, interactions between that type of cell tend to have high weights because the calculation depends on a single cell value, rather than values from two cell types. If we exclude self-loops, then for SKCM, osteoblasts, natural killer T cells, and mesenchymal stem cells (MSCs) interact with melanocytes in the top 10 scoring edges, consistent with the emerging role of these cell types in melanoma. An important role for osteoblasts is now coming to light for melanoma (Ferguson et al., 2020). Natural killer T cells are being investigated for their applicability in immunotherapy of cancers such as melanoma (Wolf et al., 2018). MSCs appear to interact with melanoma cells, as work by Zhang et al. (2017) showed the proliferation of A375 cells (a melanoma cell line) was inhibited and the cell cycle of A375 was arrested by MSCs, and cell-cell signaling related to NF-κB was down-regulated. Overall, the number of high weight edges in each tumor type did not associate with the number of samples, as might be expected (Supplementary Figure 2).

Next, we aimed at identifying which elements of the cellular communication networks might be associated with the clinical progression in particular tumor types. Disease progression and severity were examined using dichotomous values of tumor stage and PFI as described in the methods. *S*_1_ scores were calculated comparing edge weight distributions between the two clinical groups. Results were carried forward if larger than a set threshold (greater than the top 1e-6% of simulations) yielding differentially weighted edges (DWEs, Supplementary Figures 3–5).

**FIGURE 6:**
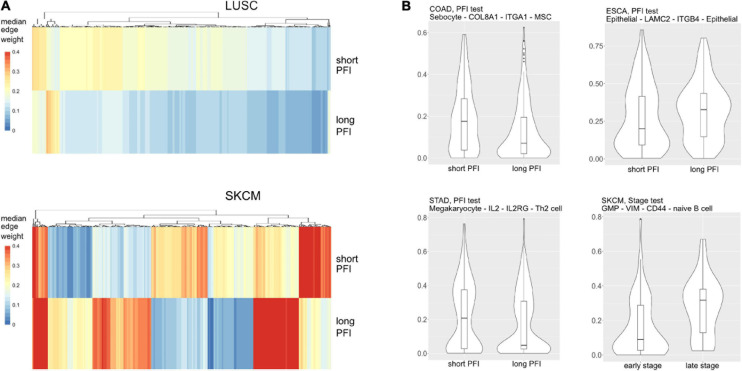
**(A)** Median values for each differentially weighted cell-cell edge (DWE) for the progression-free interval (PFI) categories (in row, DWE edges in columns). **(B)** Examples of differentially weighted edges.

Most tumor types showed DWEs for PFI, and fewer DWEs for the early to late tumor stage comparison (Supplementary Figure 5). For example, gastric adenocarcinoma (STAD) had several hundred edges in for both comparisons, while PAAD showed many fewer DWEs, and only for PFI. Figure 6 shows median edge weights between the two groups for the selected studies. Some tumor types, like SKCM, show much stronger differences between the medians, compared to the other studies like STAD, ESCA, and LUSC, which may be an indication of a stronger immune response. According to CRI-iAtlas (Eddy et al., 2020), among our example studies, SKCM has the highest estimated level of CD8 T cells and generally has a robust immune response.

The tumor stage comparison showed DWEs in 17 of 32 TCGA studies and ranged widely from 4 DWEs for MESO (mesothelioma) to over 63K DWEs for BLCA (urothelial bladder cancer adenocarcinoma). The PFI comparison showed results in 28/32 studies and ranged from 4 DWEs in READ to over 21K in LIHC. See Table 1 for DWE counts from selected studies. The studies with larger numbers of samples had random *S*_1_ distributions that were narrow compared to studies with few samples (Supplementary Figure 3), but there was not a strong association between DWE counts and sample sizes. The variation thus more likely has to do with clinical factors.

**TABLE 1:** Counts of differentially weighted edges compared to the number of samples in each study.

**Study**	**N samples**	**PFI short/long**	**PFI DWEs**	**Selected Feat**.	**Model accuracy**	**GO results?**
ESCA	170	73/97	137	36	94.7	y
STAD	409	155/231	142	78	95.1	y
PAAD	178	68/83	8	–	–	y
COAD	281	96/183	63	50	97.1	y
READ	91	16/71	4	–	–	y
SKCM	102	27/75	249	12	91.1	y
LUSC	494	193/285	304	119	98.7	y

**Study**	**N samples**	**Stage early/late**	**Stage DWEs**	**Selected Feat.**	**Model accuracy**	**GO results?**

ESCA	170	86/63	0	–	–	–
STAD	409	167/198	241	114	99.7	y
PAAD	178	142/7	0	–	–	–
COAD	281	151/118	1851	84	99.6	y
READ	91	36/44	34	18	97.5	y
SKCM	102	68/29	221	8	99	n
LUSC	494	390/89	0	–	–	–

*Study, tissue type; N samples, number of samples used; PFI short/long, number of samples in each group; PFI DWEs, number of differentially weighted edges; Model accuracy, accuracy of predicting group; GO results?, if yes, significant GO enrichments.*

**FIGURE 7:**
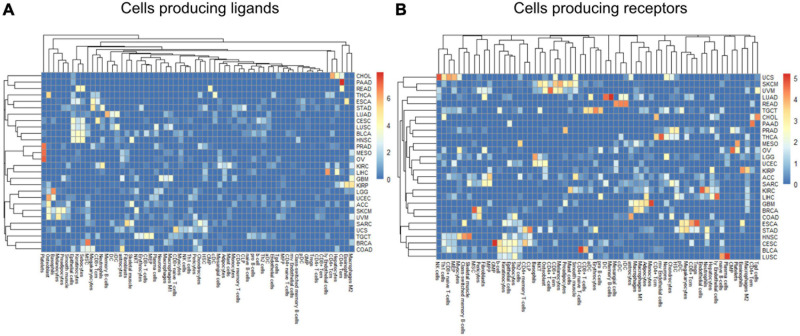
Edge member dominance in DWEs shown by log10 counts of cell types across high weight edges in ligand-bearing cells **(A)** and receptor-bearing cells **(B)**.

Within a tumor type and clinical response variable, the set of high scoring edges were usually dominated by a small number of cell-types, ligands, or receptors (Figure 7 and Supplementary Figures 6A,B). For SKCM, in the tumor stage contrast, most ligand-producing cells include GMP (granulocyte-monocyte progenitors) cells, osteoblasts, MSC cells, and Melanocytes, in order of prevalence. The number of edges starting with these four cells accounts for 53% of DWEs. Certainly, melanocytes are well known in melanoma and mesenchymal stem cells are drawn to inflammation, but the role of osteoblasts is less well documented, but still has been associated with melanoma progression (Ferguson et al., 2020).

In colorectal adenocarcinoma (COAD), for ligand-producing cells, the DWEs were dominated by astrocytes, MSCs, megakaryocytes, and sebocytes, while receptor-producing cells included astrocytes, chondrocytes, and MSCs in order of counts of DWEs. While these cells are labeled “astrocytes,” this may actually be a misnomer. These “astrocyte-like” cells that are common in the enteric nervous system of the gut are called enteric glia (Yu and Li, 2014). They both physically and molecularly resemble astrocytes of the central nervous system, but should be classified as a separate cellular entity (Jessen and Mirsky, 1983). By summarizing DWEs we can possibly categorize cancer types based on which cells are taking part in potentially active interactions.

The above-described edge dominance is related to cells (graph nodes) with high degree. In the language of graphs, the degree is the count of edges connected to a given node or vertex. In STAD where the most common cell in DWEs is the megakaryocyte, we find it is also the cell with highest degree (degree 50), followed by neutrophils (31), common lymphoid progenitors (CLP) cells (26), and erythrocytes (23) (see Supplementary Figures 6A,B).

Within the TME, communication between the multitude of cells happens simultaneously through many ligand-receptor axes. By considering a set of differentially weighted edges within a tissue type, we can construct connected networks that potentially represent multicellular communication. DWEs derived by comparing edge weights between clinical groups may indicate which parts of the cell–cell communication network shift together with disease severity.

**FIGURE 8:**
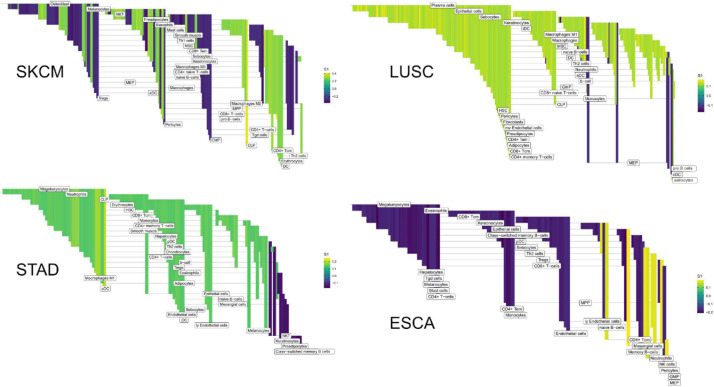
High weight edges (DWEs) from PFI contrasts form predictive connected subnetworks. Color indicates the magnitude and direction of *S*_1_ statistics (±).

**FIGURE 9:**
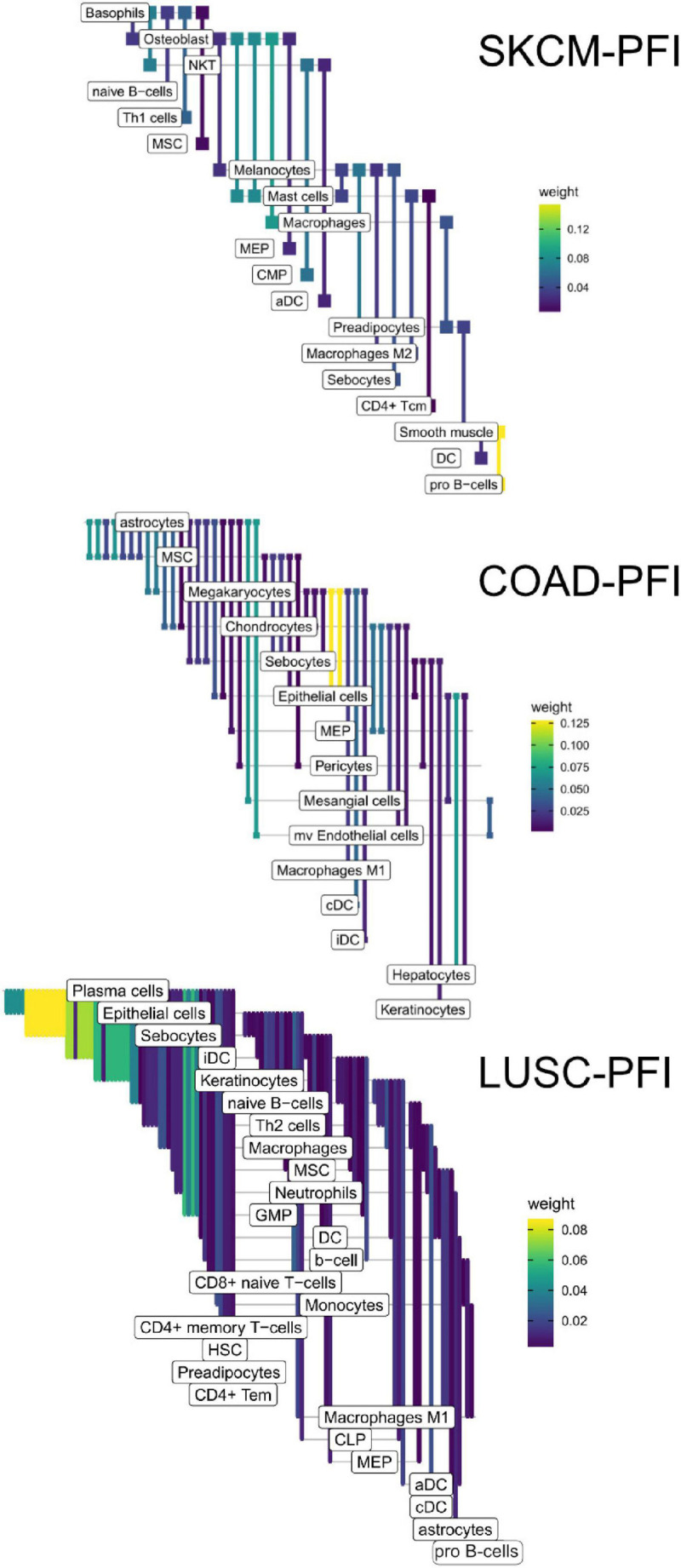
Informative edges selected by XGBoost models for prediction within study. Color indicates information gain.

The edges making up the differential networks were used to model clinical states for individual tumors (Figure 8). XGBoost models (Chen and Guestrin, 2016) were fit on each clinical feature, using edge weights as predictive variables, to infer which edges carried the most information in classification (Figure 9). The purpose of the modeling was within-data inference to determine feature importance rather than classification outside of the TCGA pan-cancer data set. The XGBoost classifiers are regularized models, not all features will be used and often only a small subset of features are utilized in the final model. We assess the relative usefulness of a feature by comparing the feature gain—the improvement in accuracy when a feature is added to a tree. All classification models had an accuracy between 91% (SKCM, PFI) and 99% (COAD, Stage). As mentioned above, there can be a high degree of correlation between edge values in a data set. During model fit, features are selected first based on improving prediction, the machine learning model accounts for correlated features by selecting the one that has best predictive power, leaving out other correlated features. That said, the number of features selected by the model is related to the correlation structure in the predictor matrix. In a set of uncorrelated features where all features add to the predictive power, all features will be selected, whereas for correlated features, only a small number will be selected.

In the COAD-PFI case, the number of features was reduced by approximately 20%, keeping 50 DWEs in the model. The STAD-PFI features were reduced by approximately 45%. Other examples are LUSC-PFI at 60% reduction, ESCA-PFI at 74%, and SKCM-PFI at 95% (12 edges selected) indicating a high degree of internal feature correlation (or lower dimensionality of edge weights).

A similar pattern was observed in the tumor stage contrasts, where SKCM-stage had a 96% reduction in features, STAD-stage 52%, READ-stage 47%. For COAD-stage, feature reduction was 95%, but attributable to the large number of starting DWEs (1,851) compared to the 84 DWEs selected. A collection of the most predictive edges is given in Table 2.

**TABLE 2:** Top five most predictive edges from XGBoost models.

**Contrast**	**Study**	**EdgeID**	**LCell**	**Ligand**	**Receptor**	**RCell**	**S1**	**Median Diff**	**Information Gain**
PFI	COAD	586640	Megakaryocytes	BMP10	ENG	Epithelial cells	0.169	0.082	0.109
PFI	COAD	50871	Astrocytes	TNC	ITGA5	mv Endothelial cells	0.168	0.061	0.069
PFI	COAD	406871	Hepatocytes	GDF2	ENG	Epithelial cells	0.168	0.082	0.067
PFI	COAD	49669	Astrocytes	EFNB1	EPHB4	Mesangial cells	0.199	0.117	0.066
PFI	COAD	632560	MEP	TIMP2	ITGB1	MEP	0.167	0.095	0.051
Stage	COAD	406579	Hepatocytes	CGN	TGFBR2	Eosinophils	–0.165	–0.077	0.043
Stage	COAD	330377	Eosinophils	LAMB3	ITGB1	Eosinophils	–0.144	–0.048	0.038
Stage	COAD	616033	Memory B-cells	BMP15	BMPR2	Epithelial cells	–0.150	–0.060	0.037
Stage	COAD	784400	NK cells	TNFSF10	TNFRSF10B	CD4+ memory T-cells	0.137	0.043	0.037
Stage	COAD	630108	MEP	B2M	KIR2DL1	iDC	0.138	0.055	0.037
PFI	ESCA	457801	Keratinocytes	GS	ADCY7	CD4+ Tcm	0.167	0.078	0.078
PFI	ESCA	182483	CD8+ Tcm	RBP3	NOTCH1	pDC	–0.184	–0.073	0.071
PFI	ESCA	1051114	Th2 cells	CALM1	GP6	naive B-cells	0.171	0.085	0.070
PFI	ESCA	658080	Mesangial cells	SPP1	CD44	Tregs	0.184	0.080	0.064
PFI	ESCA	397215	GMP	HMGB1	THBD	MEP	0.184	0.060	0.059
PFI	LUSC	879775	Plasma cells	VEGFA	ITGB1	GMP	0.120	0.047	0.041
PFI	LUSC	451902	iDC	VEGFA	ITGB1	Plasma cells	0.137	0.067	0.038
PFI	LUSC	398971	GMP	ADAM17	ITGB1	Plasma cells	0.120	0.059	0.030
PFI	LUSC	340857	Epithelial cells	COL4A6	ITGB1	CD8+ naive T-cells	0.124	0.054	0.026
PFI	LUSC	471558	Keratinocytes	THBS1	ITGA6	Plasma cells	0.120	0.068	0.025
Stage	READ	632552	MEP	TGFB3	TGFBR2	MEP	–0.267	–0.134	0.127
Stage	READ	795527	NKT	GZMB	PGRMC1	CD4+ memory T-cells	0.343	0.144	0.115
Stage	READ	402754	Hepatocytes	CGN	TGFBR2	CD4+ Tem	–0.274	–0.134	0.108
Stage	READ	808308	NKT	GZMB	IGF2R	Plasma cells	0.261	0.101	0.103
Stage	READ	800747	NKT	IL7	IL2RG	GMP	0.264	0.136	0.095
PFI	SKCM	1008243	Smooth muscle	SEMA7A	PLXNC1	pro B-cells	0.438	0.259	0.242
PFI	SKCM	517677	Macrophages	UBA52	NOTCH1	Osteoblast	–0.284	–0.145	0.200
PFI	SKCM	80934	Basophils	VIM	CD44	NKT	–0.383	–0.254	0.103
PFI	SKCM	1007915	Smooth muscle	PSAP	SORT1	Preadipocytes	0.311	0.175	0.082
PFI	SKCM	84049	Basophils	CALM1	PTPRA	Th1 cells	–0.285	–0.151	0.080
Stage	SKCM	275306	CLP	GI2	CXCR1	Osteoblast	0.353	0.176	0.207
Stage	SKCM	399084	GMP	TIMP1	CD63	Plasma cells	–0.302	–0.147	0.206
Stage	SKCM	273727	CLP	GI2	F2R	MEP	0.290	0.123	0.182
Stage	SKCM	182981	CD8+ Tcm	GI2	TBXA2R	Plasma cells	–0.283	–0.095	0.123
Stage	SKCM	397545	GMP	BST1	CAV1	MSC	–0.337	–0.194	0.109
PFI	STAD	461765	Keratinocytes	CALM3	KCNQ1	Eosinophils	–0.136	–0.067	0.062
PFI	STAD	644724	Mesangial cells	TGFB2	ACVR1	Erythrocytes	0.149	0.061	0.054
PFI	STAD	105991	CD4+ T-cells	IL1B	IL1R2	Megakaryocytes	0.134	0.081	0.047
PFI	STAD	269013	CLP	ADAM28	ITGA4	CD4+ T-cells	0.145	0.075	0.046
PFI	STAD	343620	Epithelial cells	VCAN	TLR1	CLP	0.134	0.051	0.033
Stage	STAD	128412	CD4+ Tem	CALM1	KCNQ1	Macrophages	0.140	0.058	0.057
Stage	STAD	43832	Astrocytes	FBN1	ITGB6	Epithelial cells	–0.146	–0.058	0.036
Stage	STAD	346120	Epithelial cells	LAMB1	ITGAV	Hepatocytes	–0.139	–0.066	0.035
Stage	STAD	403540	Hepatocytes	SHH	PTCH1	CD8+ T-cells	–0.138	–0.069	0.034
Stage	STAD	648983	Mesangial cells	FGB	ITGAV	Megakaryocytes	–0.140	–0.060	0.031

*Contrast, the groupwise test performed; Study, tissue type; Edge ID, BigQuery table lookup ID; LCell, cell producing ligands; Ligand, ligand gene symbol; Receptor, receptor gene symbol; R Cell, receptor producing cell; S_1_, between group S_1_ statistic; Median Diff, difference in edge weights between groups; Information Gain, Xgboost information gain after adding feature to model.*

The collection of genes from each differential network was used for GO term enrichment using the GONet tool (Pomaznoy et al., 2018). All tumor type-contrast combinations with differentially weighted edges produced enriched GO terms (FDR < 0.05, within tissue contrasts) except the SKCM-stage group, which produced no enriched terms.

Common themes included structural GO terms such as “extracellular structure organization” (for SKCM), cell–substrate adhesion (ESCA, LUSC), cell–cell adhesion (STAD), extracellular matrix (ECM) organization (LUSC, COAD, READ, and STAD). Cell migration was also a common theme with “cell migration” (STAD), “epithelial cell migration” (SKCM), and “regulation of cell migration” (LUSC, COAD/READ). Among immune related themes, GO terms included “IFNG signaling” and “antigen processing and presentation” (SKCM), “regulation of immune processes” and “IL2” (STAD), and “viral host response” (COAD/READ). See Table 3 for a summary and Supplementary Table 3 for complete results.

**TABLE 3:** Enriched GO terms.

**Tissue**	**Contrast**	**Num GOs**	**ECM**	**Migration**	**Immune**	**Immune2**
SKCM	PFI	34	Extracellular structure organization	Epithelium cell migration	IFNG signaling	Antigen processing and presentation
ESCA	PFI	3	Cell–substrate adhesion			
STAD	PFI	59	Cell–cell adhesion mediated by integrin	Cell migration	Regulation of immune system process	IL2
LUSC	PFI	39	Extracellular matrix organization	Positive regulation of cell migration		
COAD/READ	stage	85	ECM	Regulation of epithelial cell migration	Viral host response	
STAD	stage	28	ECM/adhesion	Cell migration		

*Tissue, TCGA study; Contrast, the groupwise test performed; Num GOs, number of gene ontology terms found significantly enriched; ECM, GO categories involving ECM; Migration, GO terms involving cell migration; Immune, GO terms involving immune response; Immune2, additional GO terms involving immune response.*

## Discussion

Patient outcome or response to therapy is not easily predicted by tumor stage or the somatic variations present in the tumor (Kirilovsky et al., 2016). A key factor in determining outcome will be understanding the TME, but making predictions remains difficult due to the complex nature of the disease. It has been noted that a given immune cell will have different effects on tumor progression which varies by cancer type and cell location with respect to the tumor (Fridman et al., 2012). The importance of the TME is illustrated by the “Immunoscore,” a prognostic based on the presence and density of particular immune cells in the TME, expanded to include the peripheral margin as well as tumor core. For example, the Immunoscore in colorectal cancer depends on the density of both CD3+ lymphocytes (any T cell) and specifically, CD8+ cytotoxic T cells in the tumor core and invasive margin (Pagès et al., 2018).

Along with specific cell types, previous studies have also shown that specific cellular interactions (i.e., ligand–receptor mediated interactions) within the TME have an impact on patient survival, drug response, and tumor growth. Zhou et al. (2017) described variations in ligand-receptor pair correlations when comparing cancer to normal tissue using gene expression data, the cell–cell communication structures thereby becoming a generalized phenotype for malignancy. The results showed that compared to normal tissue, the ligand-receptor correlation was reduced. The ligand-receptor pairs that commonly showed such differences across the ten tumor and matched tissue types included PLAU-ITGA5, LIPH-LPAR2, SEM14G-PLXNB2, SEMABD-TYROBP, CCL2-CCR5, CCL3-CCR5, and CGN-TYROBP.

Similar to Zhou et al. (2017), we also found associated edges enriched for related biological processes, especially to ECM organization and cell adhesion. For example, in Zhao et al., the ligand-receptor pairs COL11A1-ITGA2, COL7A1-ITGA2, MDK-GPC2, and MMP1-ITGA2 were found to be positively correlated in cancer, but uncorrelated in normal tissue. In this work, integrins and laminins generally have elevated edge weights in late tumor stages. In the PFI contrasts, except for ESCA, these edges have higher weights in the shorter PFIs, corresponding to more rapid progression.

**FIGURE 10:**
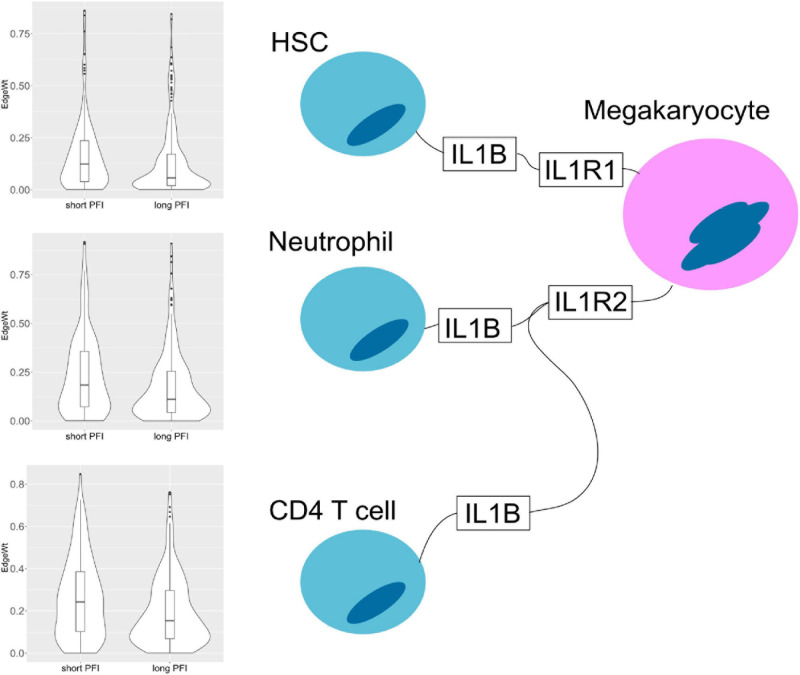
Cell–cell interaction diagram demonstrating complexity in communication with three cell types that produce the IL1B ligand that have two possible binding partners on the same receptor bearing cell. Edge weight violin plots are shown for two STAD PFI groups, short (left) and long (right) PFI.

The results also show evidence for other common tissue phenotypes such as inflammation. With SKCM and COAD, ligand producing (pro-inflammatory) M1 macrophage edges are present in the associated cell-cell networks. Also, inflammatory cytokines IL1B and IL18 are both present in the results of ESCA and STAD (Figure 10). In the tumor stage contrasts for STAD and COAD, we see Th2 and NK cells expressing inflammation related genes IL1A, IL1B, IL4, and TNF. However, we noted the absence of some well-known canonical edges such as Th1-IL12-IL12RB1-M1 macrophages. This may be due to essentially no difference, or undetectable differences in cell quantities or transcript expression between PFI groups.

Regarding cancer related cytokine networks in the literature, there is a strong emphasis on the cancer cell as a central actor. Many studies focus on cancer cell interactions in the TME. For example, cancer cells produce an overabundance of IL6 or IL10, which is associated with poor prognosis (Burkholder et al., 2014; Fisher et al., 2014; Lippitz and Harris, 2016). However, in this work, the focus has been put on the TME, excluding the cancer cell. This is largely because in performing cell deconvolution to determine the presence and quantity of different cell types in the mixed sample, reliable signatures for cancer cells are not readily available. In carcinomas, a cancer cell derives from the epithelium and in many aspects remains similar to epithelial cells. Even in single cell RNA-seq studies, it is often difficult to identify abnormal cells. Picking cells out of a mixed expression dataset using a gene expression signature remains a difficult problem.

In addition to cancer cells missing, there are also many physical aspects that cannot be addressed with the presented method, such as the rate of secretion, dynamics of binding, and cell activation. Nonetheless, by identifying the presence of edge constituents in particular TMEs, in comparison with the quantities of other constituents, it becomes more likely that communication can take place, as the presence of those constituents is a prerequisite. That is assuming the specified mRNAs are translated to the corresponding proteins. Of course, by focusing on the TME, any cell communication taking place outside of the TME is completely missed. For example, T cells and B cells are exposed to tumor derived antigens in tumor draining lymph nodes, which can potentially generate immunotolerance (Munn and Mellor, 2006).

While cancer cells are missing from the model, using cell deconvolution to identify the cell types present in a mixed sample with bulk sequencing cancer data can lead to the inclusion of surprising, unexpected, and possibly erroneous cell types. Related to ECM, cancers such as esophageal, gastric, and colorectal commonly present with metaplasia and dysplasia, a process that breaks down the structural order of a tissue (Giroux and Rustgi, 2017). Within dysplasia, unexpected cell types may be detected due to altered gene regulation which produces gene expression patterns not typically associated with healthy normal cells or possibly through transdifferentiation (Noto and Peek, 2012). For example, in pancreatic cancer, a disruption of tissue organization triggers hepatocyte differentiation in the ductal epithelium (Reddy et al., 1991). As another example from pancreatic cancer, the source of the commonly seen desmoplastic stroma (a fibrous encapsulation of the tumor) may include mesenchymal stem cells (MSCs), which possess the ability to differentiate into osteoblasts, chondrocytes, and adipocytes all of which may produce unexpected findings (Mathew et al., 2016).

Similarly to the assumptions made in communication theory, it is tempting to view communication between cells as directional, where cells produce molecules that are received by the properly paired, and expressed, receptor. There is often a sender and receiver, which makes the cell-cell networks appear to be directionally linked by molecules. While this may be useful mathematically, the reality is that there are many cases where the interaction of two cells creates a response in both cells, implying bidirectional communication. An example of this is seen with PD-L1 (programmed cell death 1) which is occasionally over expressed by tumor cells; upon binding with its cognate receptor PD1 on T cells, pathways such as stemness or chemo-resistance can be activated in cancer cells while suppressing anti-tumor immunity in T cells (Dong et al., 2018). The presented method treats cell-cell communication as undirected, but one could also use two edges in opposing directions.

In terms of the data that goes into the model, by focusing on gene expression instead of protein levels, this approach overlooks several important matters, such as the role of post-transcriptional modification. To achieve their active form, some ligands enter modification pathways after translation. For a given protein, the modification pathways available can even vary depending on cell type.

Another challenge relates to the generally weak correlation between transcript and protein levels, which is mainly due to the many levels of regulation between transcription and protein abundance. There will be cases where tight regulation leads to good correlation between transcripts and proteins, while in other cases, weak correlation may confound the results. Also, when considering protein function, some ligands and receptors are composed of several different subunits. Heterodimeric ligands can essentially create new edges if a given subunit has different binding partners (e.g., IL-12 family). Ideally, data with joint mRNA and protein abundance could potentially be used to investigate such effects.

The expression data used by the deconvolution algorithm is taken from flow-sorted cells with an assumption that we cannot identify novel (non-scaffold) edges in a tissue/cancer context. However, new data types and methods including scRNA-seq and PIC-seq will provide ways of determining new cell-cell interactions that are context specific (Giladi et al., 2020).

With the data and results publicly available in a Google BigQuery table (Supplementary Figure 8), this resource is open to researchers to explore and ask questions. It is a low-cost way (with a free tier) to achieve compute cluster performance for quickly answering questions that would otherwise be prohibitive on most in-house commodity computer systems. The BigQuery table is easily joined to clinical and molecular annotations as part of the ISB-CGC and can be worked with from R and python notebooks. With the addition of resources like GTEx, it should be possible to begin teasing apart aberrant, cancer specific interactions.

## Data Availability Statement

Publicly available datasets were analyzed in this study. This data can be found here: https://github.com/IlyaLab/Pan-Cancer-Cell-Cell-Comm-Net.

## Author Contributions

DG, BA, VT, AR, and IS conceived of the idea. DG developed the method, wrote the code, and performed the computations. DG wrote the manuscript with contributions from BA, VT, IS, and AR. IS and AR supervised the project. All authors provided critical feedback and helped shape the research, analysis and manuscript.

## Conflict of Interest

AR is a employee of Bristol Myers Squibb. The remaining authors declare that the research was conducted in the absence of any commercial or financial relationships that could be construed as a potential conflict of interest.

## Publisher’s Note

All claims expressed in this article are solely those of the authors and do not necessarily represent those of their affiliated organizations, or those of the publisher, the editors and the reviewers. Any product that may be evaluated in this article, or claim that may be made by its manufacturer, is not guaranteed or endorsed by the publisher.

## Footnotes

1
https://github.com/IlyaLab/Pan-Cancer-Cell-Cell-Comm-Net
